# C-Peptide-Based Assessment of Insulin Secretion in the Zucker Fatty Rat: A Modelistic Study

**DOI:** 10.1371/journal.pone.0125252

**Published:** 2015-05-04

**Authors:** Francesco Di Nardo, Carla E. Cogo, Emanuela Faelli, Micaela Morettini, Laura Burattini, Piero Ruggeri

**Affiliations:** 1 Department of Information Engineering, Università Politecnica delle Marche, Ancona, Italy; 2 Department of Experimental Medicine, University of Genoa, Genoa, Italy; 3 Presently at Interuniversity Centre of Bioengineering of the Human Neuromusculoskeletal System, University of Rome “Foro Italico”, Rome, Italy; Kobe University, JAPAN

## Abstract

A C-peptide-based assessment of β-cell function was performed here in the Zucker fatty rat, a suitable animal model of human metabolic syndrome. To this aim, a 90-min intravenous glucose tolerance test (IVGTT) was performed in seven Zucker fatty rats (ZFR), 7-to-9week-old, and seven age-matched Zucker lean rats (ZLR). The minimal model of C-peptide (CPMM), originally introduced for humans, was adapted to Zucker rats and then applied to interpret IVGTT data. For a comprehensive evaluation of glucose tolerance in ZFR, CPMM was applied in combination with the minimal model of glucose kinetics (GKMM). Our results showed that the present CPMM-based interpretation of data is able to: 1) provide a suitable fit of C-Peptide data; 2) achieve a satisfactory estimation of parameters of interest 3) quantify both insulin secretion by estimating the time course of pre-hepatic secretion rate, *SR(t)*, and total insulin secretion, *TIS*, and pancreatic sensitivity by means of three specific indexes of β-cell responsiveness to glucose stimulus (first-phase, *Ф_1_*, second-phase, *Ф_2_*, and steady-state, *Ф_ss_*, never assessed in Zucker rats before; 4) detect the significant enhancement of insulin secretion in the ZFR, in face of a severe insulin-resistant state, previously observed only using a purely experimental approach. Thus, the methodology presented here represents a reliable tool to assess β-cell function in the Zucker rat, and opens new possibilities for the quantification of further processes involved in glucose homeostasis such as the hepatic insulin degradation.

## Introduction

The Zucker fatty rat (ZFR) is a widely recognized experimental model of human obesity, insulin resistance and presents abnormalities similar to those observed in human metabolic syndrome [1–5]. Glucose tolerance is not regulated by the degree of insulin resistance alone, but by the capability of the β-cell to provide and keep an amount of circulating insulin able to compensate for it [6,7]. In the ZFR, for a given degree of glucose tolerance, a severe reduction of insulin sensitivity seems to be compensated by β-cell growth and a consequent increase in insulin secretion [8]. Thus, the usefulness of the ZFR as an experimental model of human metabolic dysfunctions implies the comprehensive evaluation of glucose tolerance by simultaneous quantification of physiological processes of insulin secretion, action and clearance.

Beyond direct measurements, which are hard to perform in small-sized animal, many empirical and model-based tools are available for the quantification of insulin action in the Zucker rat; in particular, our previous work confirmed the reliability of the minimal model of glucose kinetics (GKMM) in providing estimates of insulin sensitivity and glucose effectiveness [9,10].

Insulin secretion in rats and mice is usually evaluated through indexes based on a dynamic (after a glucose perturbation) insulinemia curve [11,12]. Insulinemia, however, reflects not only the effect of insulin secretion, but also insulin clearance. Studies based on insulin concentrations measured in peripheral plasma allow inferences on only post-hepatic insulin secretion process and no information on pre-hepatic insulin secretion and its extraction by the liver can be gained [13]. Recent availability of plasma C-peptide measurements in the Zucker rat [14] constitutes a turning point. C-peptide, indeed, is co-secreted with insulin in equimolar concentration, is not extracted by any appreciable extent by the liver, and has a constant metabolic clearance rate over a wide range of physiological serum concentration [9]. Thus, only through the interpretation of plasma C-peptide data, the assessment of the actual (pre-hepatic) insulin secretion process in this animal model can be achieved. Although approaches have been reported for humans, as far as we know, a C-peptide-based characterization of β-cell function in the rat is still lacking in the literature. The aim of the present study was to fill this gap providing an assessment of insulin secretion and β-cell responsiveness to glucose stimulus in the Zucker fatty rat, by means of a model-based interpretation of C-peptide plasma concentration data. To this aim, the minimal model of C-peptide secretion (CPMM), originally introduced for humans [13] and adapted here for rats, was considered.

## Materials and Methods

### Data base

This study included 14 male Zucker rats (Charles River Laboratories), divided into 2 groups: a group of 7-to-9week-old homozygous fatty rats (ZFR, fa/fa, n = 7) and a group of age-matched heterozygous lean rats (ZLR, fa/+, n = 7). All rats were housed in controlled conditions of temperature (21±1°C), humidity (60±10%) and lighting (08.00–20.00 h) and received a standard rat chow containing 0.3% sodium, with tap water *ad libitum*. The experiments were performed at 08.00 h, after a 12 h overnight fast. The animals were anesthetized with sodium pentobarbital (50 mg·kg^-1^ i.p., plus maintenance doses if necessary; Sigma Chemical, St. Louis, Missouri, USA). In our laboratory experience [15] this anesthetic has shown to be adequate, since it does not alter insulin secretion, and artefactual dose-dependent effects are not seen. The adequacy of the anesthesia was assessed by monitoring the changes in heart rate (HR) and mean arterial pressure (MAP) and by the state of the pupils. The experiments were performed in accordance with Italian national guidelines on animal experimentation (Decreto Legislativo 27/1/1992, no. 116, Attuazione della Direttiva no. 86/609/CEE in materia di protezione degli animali utilizzati a fini sperimentali o ad altri fini scientifici). The study was approved by the Ethical committee of IRCCS S. Martino-IST (Comitato per la sperimentazione etica sugli animali), Genoa, Italy (Permit n. 253). Rectal temperature was controlled and maintained at 37.5±0.5°C by a heating pad. The right femoral artery and vein were cannulated. The arterial cannula, connected to a pressure transducer (Spectramed Statham P23XL, Viggo-Spectramed, Oxnard, California, USA) provided a recording of AP through a Grass preamplifier, model 7P14A (Grass Instruments, Quincy, Massachusetts, USA). HR was monitored using a Grass tachograph (model 7P4), triggered by lead II of the electrocardiogram (ECG). The venous cannula was used for drug injection. AP, ECG and HR were digitally recorded by an A/D converter (CED Power1401, Cambridge Electronic Design, Cambridge, UK), stored on a PC and analysed by laboratory software (Spike2, CED). At the end of the experiments the animals were sacrificed by an overdose of sodium pentobarbital.

### Intravenous Glucose Tolerance Test (IVGTT)

Two basal blood samples (200 μL) were taken from the arterial catheter at—5 and—2 min before glucose injection. A glucose bolus of 400 mg·kg^-1^ was then injected over 1 min into the femoral vein (conventional time-zero). Ten additional blood samples (100 μL each) were collected at 1, 2, 3, 5, 8, 15, 25, 40, 70 and 90 min after the injection, for the measure of glucose, insulin and C-peptide concentration. Plasma volume was replaced by controlled normal saline infusion.

### Assays

Blood was promptly centrifuged and glucose immediately measured with the glucose oxidase method using an automated glucose analyser. The remaining plasma was stored at -80°C for later insulin determination. Insulin and C-peptide were measured with commercially available rat insulin and rat C-peptide ELISA kits (Mercodia, Uppsala, Sweden). The sensitivity of the insulin assay is 0.07 g·L^-1^, with an inter-and intra- assay precision of 3.3 and 1.8 respectively. The sensitivity of the C-peptide assay is 27.5 pmol·L^-1^ with an inter-and intra- assay precision of 2.9 and 4.4 respectively.

### Assessment of insulin sensitivity and glucose effectiveness

The GKMM [16] was applied to fit glycemia data, using insulinemia data as model input. This procedure, described in detail in our previous work [17], yielded quantitative information on well-established indexes of whole-body insulin sensitivity, *S*_*I*_, and glucose effectiveness, *S*_*G*_.

*S*_*I*_ measures the ability of insulin to enhance plasma glucose disappearance and to inhibit hepatic glucose production. This index is useful to discriminate insulin resistance state, defined as abnormally low insulin sensitivity. *S*_*G*_ quantifies the ability of glucose per se to enhance its rate of disappearance and to inhibit hepatic glucose production. The measurement units are dL·kg^-1^·min^-1^/(μU·mL^-1^) for *S*_*I*_ and dL·kg^-1^·min^-1^ for *S*_*G*_.

### Assessment of β-cell responsiveness

The minimal model of C-peptide secretion (CPMM), originally introduced for humans by Cobelli & Pacini [13], was applied to fit C-peptide data using glycemia data as model input. CPMM features the two components of insulin secretion in response to glucose challenge. The former represents the secretion of promptly releasable insulin and is quantified by the index *Φ*_*1*_, which measures the incremental amount of C-peptide released during the first phase of β-cell response to the increment of plasma glucose concentration. *Φ*_*1*_ is referred to as the first-phase β-cell responsiveness to glucose stimulus. The latter relates to the provision of new insulin to the releasable pool and is characterized by *Φ*_*2*_, which quantifies the stimulatory effect of glucose concentration on provision into the β-cells and release of new insulin. *Φ*_*2*_ is referred to as the second-phase β-cell responsiveness to glucose stimulus. A third index, *Φ*_*ss*_, represents the β-cell secretory response to steady-state glycemia. *Φ*_*ss*_ is referred to as the steady-state β-cell responsiveness to glucose stimulus. CPMM is able to provide two further quantities useful to quantify the secretion process: the time course of above steady-state C-peptide secretion rate, *SR(t)*, and the total amount of C-peptide secreted by the β-cells, *TIS*. The measurement units are (pmol/L C-peptide)/(mmol/L glucose) for *Φ*_*1*_, min^-2^·(pmol/L C-peptide)/(mmol/L glucose) for *Φ*_*2*_, min^-1^·(pmol/L C-peptide)/(mmol/L glucose) for *Φ*_*ss*_, (min^-1^·pmol/L) for *SR(t)* and (pmol/L) for *TIS*.

The CPMM parameters [13] (*k*_*01*_, *k*_*12*_, *k*_*21*_, γ, *h*, and *CP*_*0*_) are defined in Appendix 1. For clarity, mathematical definition of model indexes and model equations are also reported in Appendix 1.

### Parameter Estimation

Free CPMM parameters are *k*_*01*_, *k*_*12*_ and *k*_*21*_ for kinetics; γ, *h*, and *CP*_*0*_ for secretion (Appendix 1). Although it was reported that all these are a priori uniquely identifiable [12], it was demonstrated that, for reliable numerical identification, knowledge of population parameters for *k*_*01*_, *k*_*12*_ and *k*_*21*_ is required [18]. Since population values of these parameters are not available for rats, the mean values assessed in mice by Ahrèn et al. [19], following intravenous administration of human C-peptide (i.e. *k*_*01*_ = 0.195 min^-1^, *k*_*12*_ = 0.133 min^-1^ and *k*_*21*_ = 0.183 min^-1^) were adopted here. Moreover, the *h* parameter was set to the steady-state (end-test) glycemia value, *G*_*ss*_. Thus, secretion parameters γ, and *CP*_*0*_ were estimated in each rat by fitting to measured C-peptide data. A non-linear least squares estimation technique [20] implemented in SAAM II software [21] was used. Glucose data were assumed as error-free model input and were linearly interpolated for the simulation. Errors in C-peptide measurements were assumed to be uncorrelated, Gaussian, zero mean. Since the weights of the data (i.e., the error affecting the data) were not exactly known, a relative weighting procedure, where the weighting constant was estimated directly from the data themselves, was performed. Precision of parameter estimates was expressed as percent coefficient of variation, CV% = (*SD*_*pi*_ *∕ pi*)·100 where *SD*_*pi*_ is the parameter standard deviation derived from the inverse of the Fisher information matrix and *p*_*i*_ is the related parameter estimate [20]. The responsiveness indexes *Φ*_*1*_, *Φ*_*2*_, and *Φ*_*ss*_ and the time-course of the C-peptide secretion rate for unit of distribution volume (total, *TSR(t)*, and above steady-state, *SR(t)*) were subsequently computed for individual cases; when needed, their precision was evaluated by the error propagation rule. The integral of *TSR(t)* over 90-min IVGTT yielded the total amount, *TIS*, of C-peptide secreted by the β-cells, for unit of distribution volume.

### Sensitivity analysis

The robustness of the presented method was tested by analyzing the sensitivity of the CPMM-estimated insulin responsiveness indexes, *Φ*_*1*_, *Φ*_*2*_, and *Φ*_*ss*_, to an “en bloc” ±20% variation of kinetic parameters *k*_*01*_, *k*_*12*_ and *k*_*21*_. *Φ*_*1*_^*+20*^, *Φ*_*2*_^*+20*^, and *Φ*_*ss*_^*+20*^ and *Φ*_*1*_^*-20*^, *Φ*_*2*_^*-20*^, and *Φ*_*ss*_^*-20*^ were referred to the responsiveness indexes estimated after +20% and -20% variation of kinetic parameters, respectively.

### Statistical analysis

The Lilliefors test (suitable for small samples) was used to evaluate the hypothesis that each data vector or parameter vector had a normal distribution with unspecified mean and variance. Comparisons between two groups of normally distributed samples were performed with two-tailed, non-paired Student’s *t* test; Wilcoxon rank sum test was used to compare samples which were not normally distributed. To quantify the linear regression analysis, Pearson’s product-moment correlation coefficient and Spearman's rank correlation coefficient were used for normally and non-normally distributed populations, respectively.

In order to determine the retrospective power of an observed effect based on the sample size and parameter estimates derived from the data set, a post-hoc statistical power analysis has been performed. The analysis was performed using G*Power 3.1, a free general power analysis tool [22]. The power was computed in relation to the hypothesis test used.

Statistical significance was set at 5% level for every test used in the present study.

## Results

**Fig 1:**
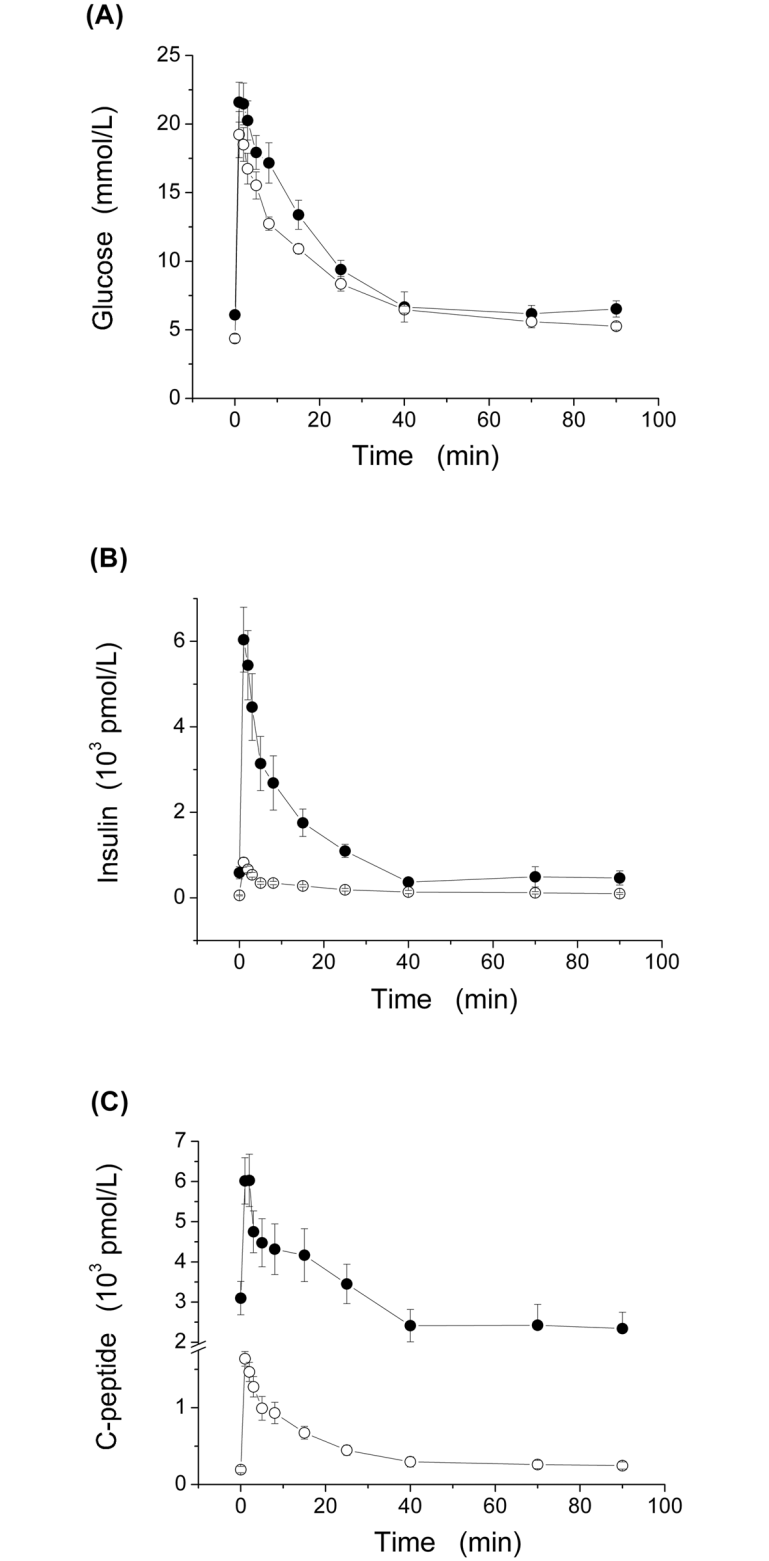
Time course of plasma glucose (*G(t)*, panel A) insulin (*I(t)*, panel B) and C-peptide (*CP(t)*, panel C) concentrations during 90-min IVGTT in our ZFR group (n = 7, closed circles) and ZLR group (n = 7, open circles). Values are mean ± SE.

Compared to the age-matched ZLR group, the ZFR group was characterized by having, on average, significantly higher body weight (BW), systolic (SAP), diastolic (DAP) and mean (MAP) aortic pressure, and fasting plasma concentration of glucose, insulin and C-peptide (Table 1). The time courses of mean (±SE) glucose, *G(t)*, insulin *I(t)* and C-peptide, *CP(t)*, plasma levels, throughout the entire IVGTT, are represented in Fig 1A, 1B and 1C, respectively. A significant increase was observed in the ZFR group, compared with the ZLR group, throughout the entire IVGTT for *CP(t)*, between 1 and 70 min for *I(t)*, and between 8 and 15 min for *G(t)*.

**Table 1:** Characteristics of our groups of Zucker rats.

Variable	ZLR(n = 7)	ZFR(n = 7)	Statistics
Age (wk)	8.1 ± 0.3	8.4 ± 0.2	NS**
BW (g)	225 ± 12	278 ±8	*P* < 0.005*
MAP (mmHg)	98 ± 2	107 ± 1	*P* < 0.005*
SAP (mmHg)	113 ± 3	124 ± 3	*P* < 0.05*
DAP (mmHg)	81.8 ± 1.2	90.4± 1.1	*P* < 0.001**
Glycemia (mmol·L^-1^)	4.36 ± 0.31	6.09 ± 0.19	*P* < 0.001*
Insulinemia (pmol·L^-1^)	55.3 ± 11.1	584 ± 142	*P* < 0.005*
C-peptide (pmol·L^-1^)	193 ± 41	3095 ± 416	*P* < 0.001**
*IAUC* (10^3^ pmol/L in 90 min)	16.5 ± 1.8	93.5 ± 14.8	*P* < 0.001*
*CPAUC* (10^3^ pmol/L in 90 min)	39.1 ± 4.8	270 ± 42	*P* < 0.001*

Values are means ±SE. BW, body weight; MAP, mean arterial pressure; SAP, systolic arterial pressure; DAP, diastolic arterial pressure. *IAUC* and *CPAUC*, area under insulin and C-peptide curves, respectively, computed by the trapezoidal rule; NS, not significant;

* Unpaired Student’s t-test;

** Wilcoxon rank sum test. (min-1·pmol/L), steady-state secretion rate.

**Fig 2:**
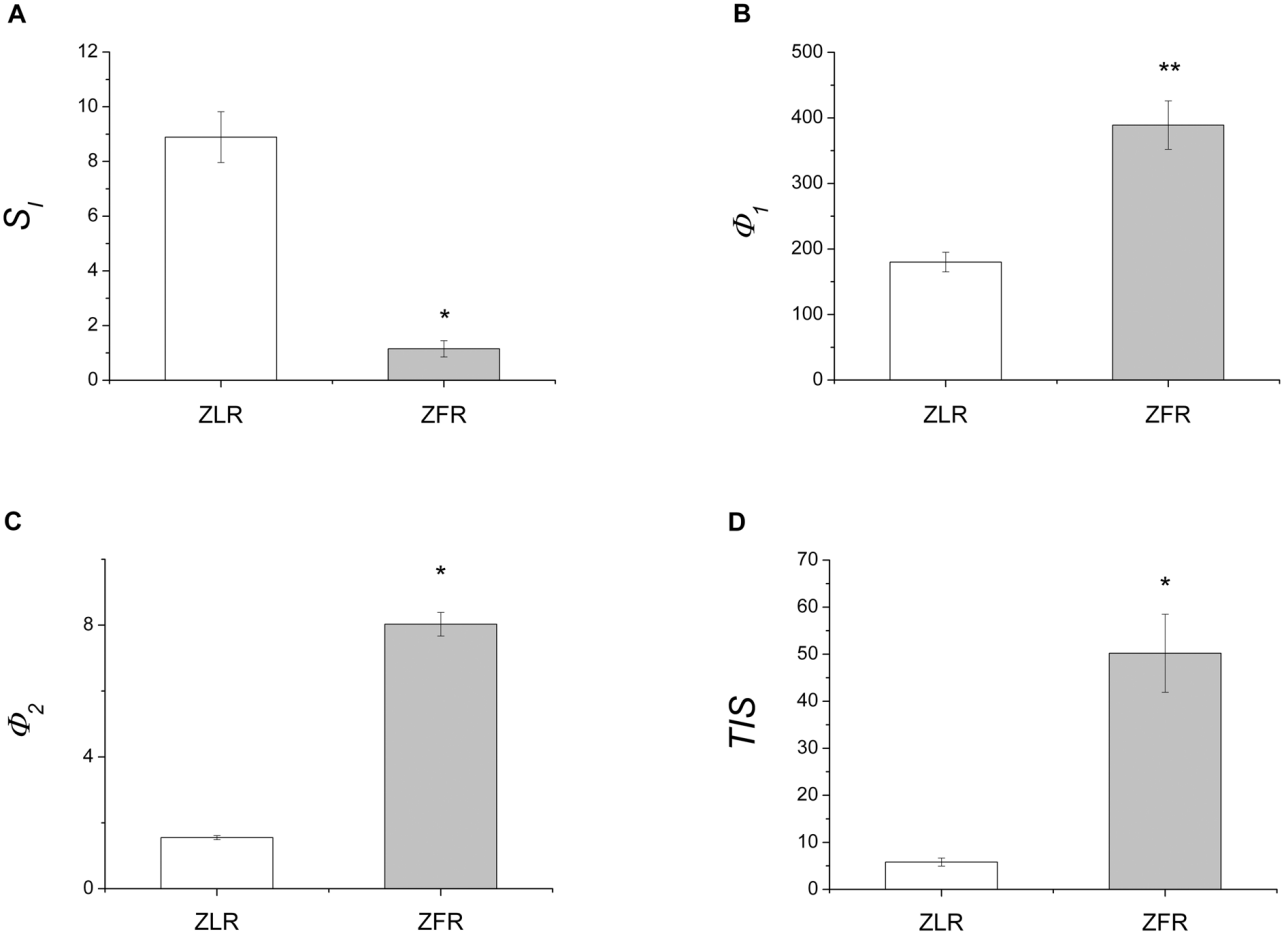
IVGTT-based mean (±SE) values of model predicted: whole-body insulin sensitivity (*S*_*I*_, panel A); first-phase β-cell responsiveness to glucose stimulus (*Φ*_*1*_, panel B); second-phase β-cell responsiveness to glucose stimulus (*Φ*_*2*_, panel C) and total insulin secretion, for unit of distribution volume (*TIS*, panel D) in ZLR (open bar) and ZFR (shaded bar) groups. Measure units are: 10^-4^·dL·kg^-1^·min^-1^/(μU·mL^-1^) for *S*_*I*_; (pmol/L C-peptide)/(mmol/L glucose) for *Φ*_*1*_; 10^-1^·min^-2^·(pmol/L C-peptide)/(mmol/L glucose) for *Φ*_*2*_; and 10^3^·pmol/L for *TIS*. * p<0.001 and ** p<0.05 in comparing ZFR and ZLR groups.

Estimates of *S*_*I*_ in individual rats are given in Table 2 for ZLRs and ZFRs. Estimates of *S*_*G*_ in individual rats have been reported in S1 Table. A significant 87% reduction in mean estimates of *S*_*I*_, provided by the interpretation of IVGTT data by the GKMM, was detected in the ZFR group (Fig 2A), compared to the ZLR group, whereas no significant differences were found in *S*_*G*_ between ZLR (7.74±1.40×10^-2^·dL·kg^-1^·min^-1^) and ZFR (5.49±0.77×10^-2^·dL·kg^-1^·min^-1^) groups (S1 Table). The estimated precision from the least squares procedure, expressed by mean (±SE) percent coefficient of variation, CV%, was 17.3±2.8 for *S*_*I*_ and 15.6±3.6 for *S*_*G*_, over the ZLR group and 24.0±9.5 for *S*_*I*_ and 25.5±10.8.4 for *S*_*G*_, over the ZFR group.

**Table 2:** Parameters of insulin action and secretion in ZLR and ZFR groups.

ZLR	*S*_*I*_	*Φ*_*1*_	*Φ*_*2*_	*Φ*_*ss*_
1	10.7 (15.3)	194 (14.7)	1.35 (33.3)	6.98
2	6.99 (11.4)	197 (14.3)	1.72 (29.7)	5.20
3	4.19 (25.3)	121 (21.6)	1.44 (33.5)	3.74
4	10.6 (29.7)	157 (14.8)	1.49 (30.7)	6.90
5	9.83 (8.6)	147 (12.8)	1.77 (29.3)	7.27
6	10.0 (14.8)	233 (29.0)	1.50 (29.3)	14.6
7	10.5 (16.1)	210 (17.6)	1.58 (32.3)	12.2
ZFR	*S*_*I*_	*Φ*_*1*_	*Φ*_*2*_	*Φ*_*ss*_
1	1.6 (17.5)	268 (6.8)	7.40 (20.8)	18.9
2	0.91 (4.0)	295 (8.8)	9.97 (22.5)	58.3
3	0.12 (77.0)	454 (20.3)	7.80 (30.4)	91.5
4	0.45 (22.2)	363 (19.9)	7.38 (33.5)	101
5	2.54 (16.6)	376 (20.1)	7.47 (33.1)	138
6	1.47 (30.4)	411 (19.5)	7.59 (32.5)	118
7	0.95 (5.3)	556 (17.7)	8.58 (28.8)	58.6

*S*_*I*_ (10^-4^ dL·kg^-1^·min^-1^ /(μU·mL^-1^)), whole-body insulin sensitivity; *Φ*_1_((pmol/L C-peptide)/(mmol/L glucose)), first-phase responsiveness index; *Φ*_*2*_ (10^-1^ min^-2^·(pmol/L C-peptide)/(mmol/L glucose)), second-phase responsiveness index; *Φ*_*ss*_ (min^-1^·(pmol/L C-peptide)/(mmol/L glucose)), steady-state responsiveness index. All the parameters and indexes were estimated or computed by CPMM and IVGTT, except for *S*_*I*_, which was estimated by GKMM and IVGTT. The percent coefficient of variation of the estimates (CV%) was given in parentheses, when available.

**Fig 3:**
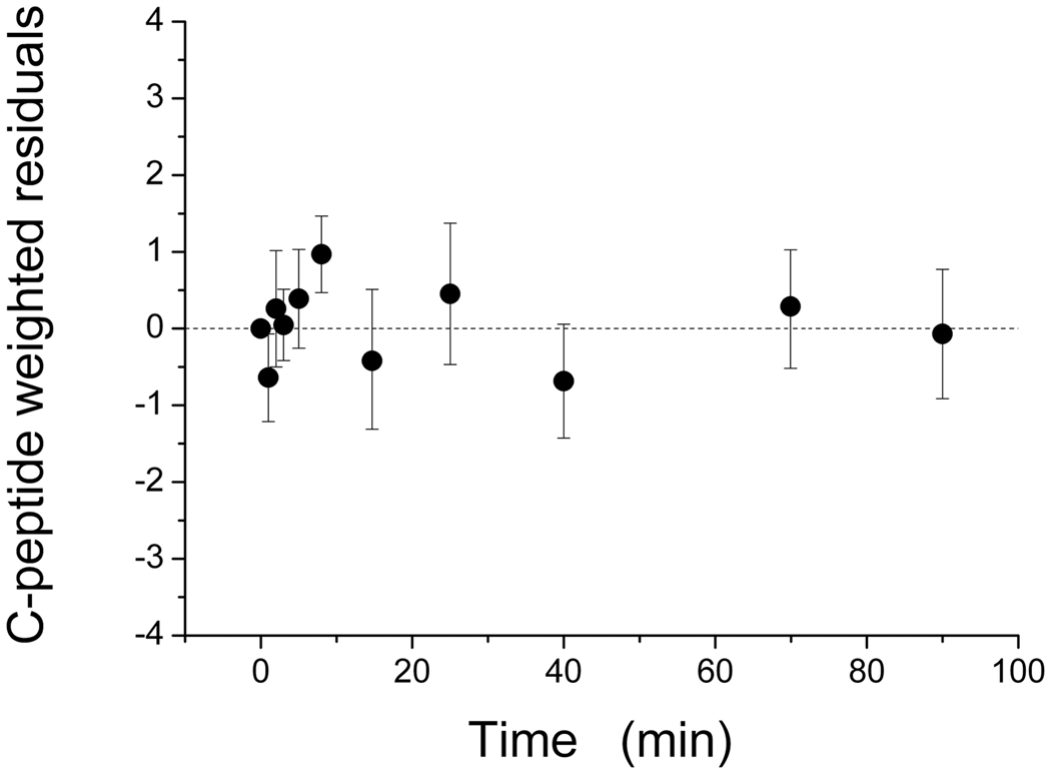
Mean (±SD) weighted residual over all our fourteen Zucker rats (7 ZFRs and 7 ZLRs) provided by fitting the CPMM output to C-peptide data.

**Fig 4:**
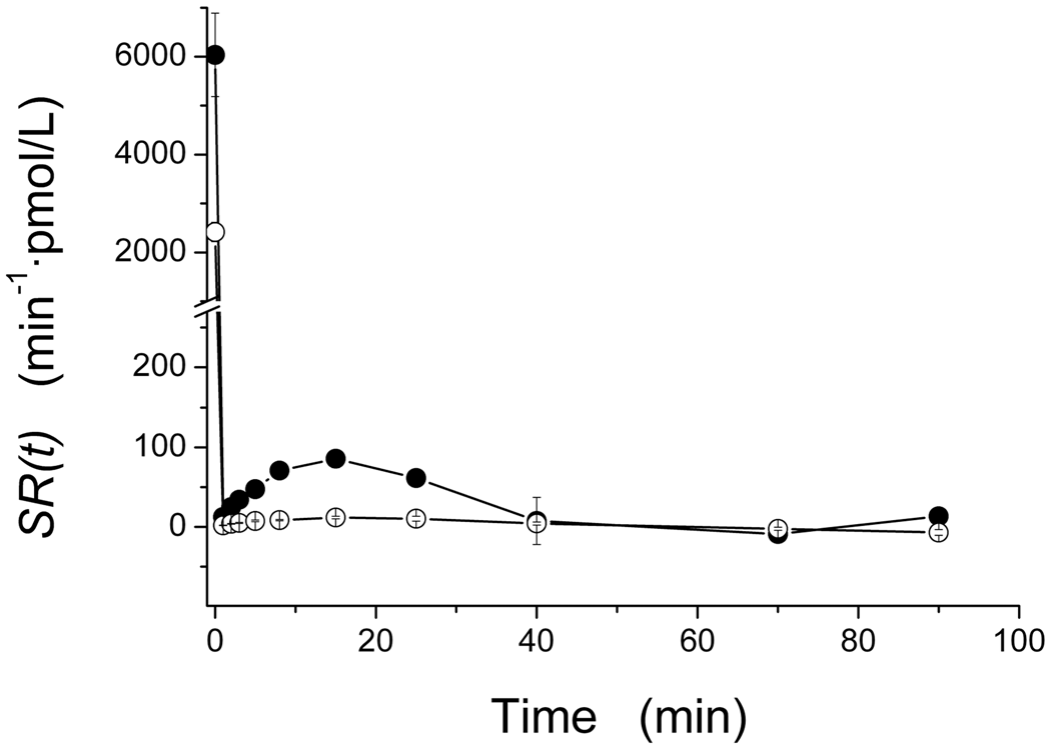
Time course of above steady-state insulin secretion rate, *SR(t)*, during 90-min IVGTT in our ZFR group (n = 7, closed circles) and ZLR group (n = 7, open circles). Values are mean ± SE.

Quantification of β-cell function was achieved by the interpretation of C-peptide data by the minimal model of C-peptide secretion (CPMM). CPMM provided a good fit to C-peptide data, as judged from the average weighted residuals (over all fourteen rats, Fig 3) which showed no systematic deviation from zero and were within the range [-1, +1]. This behavior is consistent with the hypothesis that the measurement error was a random variable normally distributed, around zero. Estimates of main CPMM parameters and steady-state indexes in individual rats are given in Table 2 for ZLRs and ZFRs. Precision is given in parentheses. The individual estimates of the other model parameters have been reported in S1 Table. A significant difference in mean estimates of *Φ*_*1*_ (116%, Fig 2B), *Φ*_*2*_ (418%, Fig 2C) and *Φ*_*ss*_ (927%) was detected in the ZFR group, compared with the ZLR group, indicating an overall enhanced responsiveness of β-cell secretion to glucose stimulus. Mean steady-state secretion rate, *SR*_*ss*_ (min^-1^·pmol/L), was significantly higher in the ZFR group (498±85) compared to the ZLR group (47.8±9.6). Mean above steady-state secretion rate, *SR(t)*, showed a significant increase in our ZFR group, between 0 and 25 min (Fig 4). Thus, total insulin secretion rate *TSR(t)* (*TSR(t) = SR(t) + SR*_*SS*_, see Eq A8 in the Appendix 1), was significantly higher in the ZFR group throughout the entire IVGTT. Integration of *TSR(t)*, over 90 min yielded a significant 761% increase in total insulin secretion per unit of distribution volume, *TIS*, in the ZFR group (Fig 2D).

The physiological process of insulin clearance was evaluated here by *IC* index, calculated as the ratio between the integral of *SR(t)* and the area under the insulin curve (*IAUC*). No significant alteration in mean *IC* between the two groups (69.1±16.3 vs. 84.2±9.4 min^-1^, *P*>0.05) indicated that the ability of the ZFR to remove insulin from the circulation remained unchanged.

The product of insulin sensitivity with an index of C-peptide derived β-cell function (sometimes termed Adaptation Index) provides figures of the capacity of the β-cell to adapt its secretion to the changes in insulin resistance [23]. In the present paper, the Adaptation Index has been computed as *AI* = *S*_*I*_ · peak C-peptide. A significant decrease in mean (±SE) estimates of *AI* (2.24±0.29 vs. 1.03±0.27 · 10^-1^·dL·kg^-1^·min^-1^, 54%) was detected in the ZFR group, compared with the ZLR group.

Sensitivity analysis to an “en bloc” ±20% variation of kinetic parameters, performed to test the robustness of the method, resulted in variations of ±13% in mean *Φ*_*1*_, ±3% in mean *Φ*_*2*_, and ±20% in mean *Φ*_*ss*_. Differences between mean estimates of the parameters before and after a ±20% variation of kinetic parameters were not statistically significant. Thus, the significant increase of *Φ*_*1*_, *Φ*_*2*_, and *Φ*_*ss*_ values found in ZFR-group, with respect to ZLR-group, remained substantially unaltered to a both +20% and -20% variation of kinetic parameters. Linear regression analysis showed a strong correlation between estimated values of *Φ*_*1*_, *Φ*_*2*_, and *Φ*_*ss*_ and corresponding estimates of *Φ*_*1*_^*+20*^ (Pearson: R = 0.99, *P<*0.0001), *Φ*_*2*_^*+20*^ (Spearman: R = 0.96, *P<*0.0005), and *Φ*_*ss*_^*+20*^ (Spearman: R = 1, *P<*0.0001) and *Φ*_*1*_^*-20*^ (Pearson: R = 0.99, *P<*0.0001), *Φ*_*2*_^*-20*^ (Spearman: R = 0.99, *P<*0.0001), and *Φ*_*ss*_^*-20*^ (Spearman: R = 1, *P<*0.0001), respectively.

Power analysis was performed to test the reliability of the significant differences detected between populations: the power of tests was not lower than 0.95.

## Discussion

The present study was designed to provide a methodology for the C-peptide-based assessment of insulin secretion and β-cell responsiveness to glucose load in the Zucker fatty rat, a suitable experimental model of human obesity and insulin resistance. To this aim, the minimal model of C-peptide secretion (CPMM), originally introduced for humans [13], was adapted here for Zucker rats. Our study involved seven Zucker fatty rats (ZFR group) and seven Zucker lean rats (ZLR group), which underwent a 90-min intravenous glucose tolerance test (IVGTT) to measure glucose, insulin and C-peptide plasma concentration. This study was performed on 7-to-9-week-old rats because we previously demonstrated that in ZFR insulin sensitivity is fully established at the age of 7 weeks and remains practically unaltered until at least the 16^th^ week [24]. On average, our ZFR group, compared with the age-matched ZLR group, presented significantly higher body weight (BW), systolic (SAP), diastolic (DAP) and mean (MAP) aortic pressure, and fasting plasma concentration of glucose, insulin and C-peptide (Table 1).

### Methodological considerations

The β-cell secretory response to glucose stimulus was assessed by adapting to Zucker rats the minimal modeling of IVGTT C-peptide data, widely used in humans [13,18,25,26]. Although all model parameters are uniquely identifiable [27], numerical identification of the model requires the knowledge of C-peptide kinetic parameters (*k*_*01*_, *k*_*12*_ and *k*_*21*_) to avoid a bias that may affect parameter estimates, due to undesired compensations in the simultaneous assessment of kinetics and secretion from the same set of C-peptide data [18]. In the absence of direct measurements in the rat, kinetic parameters were given the mean values assessed in mice by Ahrèn et al. [19], following intravenous administration of human C-peptide. Despite these values being not independently validated in our population, two aspects support the plausibility of our assumption: 1) Ahrèn et al. [19] found that variations in the parameters of C-peptide kinetics within their animal population were small and body weight had no influence in spite of the large variability among animals; 2) C-peptide kinetics, estimated in mice, was comparable to that previously reported in humans [28] and dogs [29]. This suggests a small variability among species. The secretory parameter *h* (defined as the glucose threshold level above which second-phase secretion starts) was given end-test glycemia value, *G*_*ss*_; equivalence between *h* and *G*_*ss*_ values was also reported in model-based study on humans [26,30]. These assumptions enabled us to achieve a reliable description of β-cell secretion in Zucker rats by the estimation of only two fundamental parameters (γ and *CP*_*0*_). Precision of the estimation process improves by increasing the number of experimental samples and/or decreasing the number of parameters to assess [31]. Because the IVGTT protocol in the Zucker rat consisted of a limited number of blood samples (ten samples vs. twenty-four samples collected in humans), a low number of parameters was the only way to reduce the estimation error.

Mean weighted residuals, reported in Fig 3 for all fourteen rats, indicate that CPMM provides a suitable fit of C-peptide data. Parameters of interest, such as first phase, *Φ*_*1*_and second phase, *Φ*_*2*_, β-cell responsiveness to glucose stimulus were estimated with mean (over all fourteen rats) CV% < 20% and < 30%, respectively. The detailed individual results (Tables 1 and 2) and the significant correlation between *Φ*_*1*_ and *Φ*_*2*_ in all fourteen rats (Spearman: R = 0.82; *P*<0.0005) show that the parameter distributions are fair, supporting the conclusion that parameter estimation is satisfactory. Robustness of the estimates of these glucose-stimulated parameters, as well as the computed value of *Φ*_*ss*_, is supported by the sensitivity analysis to ±20% variation of kinetic parameters. Indeed, our sensitivity analysis: 1) detected no significant alteration of mean *Φ*_*1*_, *Φ*_*2*_ and *Φ*_*ss*_; 2) provided correlation coefficients close to 1; and 3) confirmed the ability of these indexes to discriminate a significant increase of β-cell responsiveness in ZFR. Reliability of our estimation procedure is supported by the fact that mean insulin secretion patterns *SR(t)* (Fig 4) are characterized by an initial sharp peak and a subsequent rapid fall to a minimum followed by a rise, before the final settlement about the end-test steady state. This description of the insulin secretion rate is comparable with those reported for humans [18,30,32].

### Physiological considerations

In our ZFR group, a significant difference in mean *Φ*_*1*_ (116%, Fig 2B) and *Φ*_*2*_ (418%, Fig 2C), with respect to the ZLR group, suggested an overall enhancement in β-cell responsiveness to glucose stimulus, which is stressed by a significant increase in mean steady-state responsiveness index, *Φ*_*ss*_ (927%). The enhanced first and second phase β-cell responsiveness is likely to be the cause of the significant increase of the above steady-state C-peptide secretion rate, *SR(t)*, detected during the first 25 minutes of our IVGTT test (Fig 4). The significant increment of the total amount, *TIS*, of C-peptide secreted by the β-cells, for unit of distribution volume (761%, Fig 2D) is consistent with the significantly higher (591%) value of the mean (±SE) area under the C-peptide curve, *CPAUC*, observed in the ZFR group compared to ZLR group (Table 1).

For an appropriate characterization of glucose tolerance, β-cell secretory response to glucose stimulus should be evaluated in face of insulin sensitivity [6,7]. Thus, a model-based index of insulin sensitivity was assessed in the present study. Our assessment of glucose kinetics by the minimal model and IVGTT technique showed a significant 87% reduction of *S*_*I*_ in our ZFR group, compared with ZLR group (Fig 2A). This confirmed the presence of a severe insulin-resistant state in the ZFR, in accordance with foregoing reports [24,33,34]. In agreement with our previous results [10,24], no significant change in *S*_*G*_ estimates showed that, in the ZFR, the capability of glucose *per se* to enhance glucose disposal and suppress endogenous glucose production, at basal insulin, is not significantly different from that of the ZLR.

These findings indicate that the ZFR enhances the β-cell responsiveness and secretion, in the attempt of compensating the severe insulin resistant state. Despite that, the ZFR showed a mean significant increase in steady-state glycemia, compared to the ZLR (Table 1). This suggests that, in the presence of no alterations of insulin clearance between groups, it is likely that the compensatory enhancement of β-cell secretion in the ZFR is only partially balancing the insulin resistance, not being able to maintain the steady-state glycemia at the same level as observed in the ZLR. The significant decrease detected in mean Adaptation Index (*AI*) seems to confirm that this compensation is only partial and results in a limitation of glycemia increase. However, at this stage this does not necessarily represent a compensation defect. The agreement of the findings achieved in the present study, using a model-based approach, with the findings achieved by Jetton et al. [8], using a purely experimental approach, represents a further support for the reliability of our estimation procedure.

Adapting and validating the CPMM allowed to quantify both insulin secretion by means of the time course of secretion rate (*SR(t)*) and β-cell responsiveness by means of three specific indexes (*Φ*_*1*_, *Φ*_*2*_ and *Φ*_*ss*_), never assessed in rats before. The availability of a quantitative tool to reliably analyse the β-cell function opens new possibilities for a deeper quantification of glucose tolerance in Zucker rats. Combined with a model of insulin kinetics, indeed, CPMM could be able to provide a model-based estimate of the hepatic insulin degradation process, whose role in modulating insulin delivery to peripheral circulation, in rats (and also in insulin-resistant humans) is not yet fully understood. Possible further developments of the present research will be focused on this issue.

## Conclusion

In the present study, a C-peptide-based assessment of β-cell function as it relates to insulin resistance was achieved for the first time in the Zucker fatty rat (ZFR). A methodology based on the minimal model of C-peptide (CPMM), originally introduced for humans, was adapted here in Zucker rats. For a comprehensive evaluation of glucose tolerance in ZFR, CPMM was applied in combination with the minimal model of glucose kinetics (GKMM). Estimates of first-phase, *Φ*_*1*_, second-phase, *Φ*_*2*_, and steady-state, *Φ*_*ss*_, indexes of β-cell responsiveness to glucose stimulus, pre-hepatic insulin secretion rate, *SR(t)*, and total insulin secretion, *TIS*, were assessed in the young ZFR, compared to an age-matched ZLR control strain. Thus, our results demonstrated that the present model-based approach provides a suitable fit of C-Peptide data, achieves a satisfactory estimation of parameters of interest, and is able to detect the significant enhancement of insulin secretion in the ZFR, in face of a severe insulin-resistant state, previously observed only using a purely experimental approach.

## Appendix 1

### Model Equations

The minimal model of C-peptide secretion (CPMM) [13] accounts for the effect of plasma glucose concentration on biphasic C-peptide secretion in an accessible compartment, which represents the plasma. Biphasic secretion rate, *SR(t)* (min^-1^·pmol/L), is defined as:

$$SR\left(t\right)\;=\;SR_{1}\left(t\right)+\;SR_{2}\left(t\right)$$ (A1)

The *SR*_*1*_ *(t)* (min^-1^·pmol/L) is described by an impulsive C-peptide release (above steady-state) of amplitude *CP*_*0*_ at time 0^+^ (*δ(t)* is the Dirac function):

$$SR_{1}\left(t\right)\;=\;CP_{0}\cdot \delta \left(t\right)$$ (A2)

The *SR*_*2*_ *(t)* (min^-1^·pmol/L) represents the slower secretion phase, following the provision of new C-peptide to the β-cells [13].

$$SR_{2}\left(t\right)\;=\;\gamma \cdot \left(G\left(t\right)-h\right)\cdot t$$ (A3)

where *G(t)—h* (mmol/L) is the deviation of plasma glucose concentration *G(t)* from a threshold level, *h*; *γ* (min^-2^·(pmol/L C-peptide)/(mmol/L glucose)) is a constant and *t* is the time interval which follows the glucose injection.

C-peptide kinetics are described by the two-compartment model, originally proposed for humans [35]:

$$\begin{array}{l} \dot{CP_{1}}\left(t\right)\;=\;-\left[k_{01}+k_{21}\right]\cdot CP_{1}\left(t\right)+\;k_{12}\cdot CP_{2}\left(t\right)+SR\left(t\right)\;CP_{1}\left(0\right)\;=\;0\\ \dot{CP_{2}}\left(t\right)\;=\;k_{21}\cdot CP_{1}\left(t\right)-\;k_{12}\cdot CP_{2}\left(t\right)\;CP_{2}\left(0\right)\;=\;0 \end{array}$$ (A4)

Where *CP*_*1*_ *(t)* and *CP*_*2*_ *(t)* are the C-peptide concentration above steady-state level (pmol/L) in the accessible and peripheral compartment, respectively. Kinetic parameters (min^-1^) are denoted by the constants *k*_*01*_, *k*_*12*_ and *k*_*21*_.

The CPMM provides two indexes, *Φ*_*1*_ and *Φ*_*2*_, respectively related to the first and the second phase of β-cell responsiveness to glucose stimulus. The index *Φ*_*1*_ ((pmol/L C-peptide)/(mmol/L glucose)) measures the incremental amount of C-peptide, *CP*_*0*_, (per unit volume of compartment 1) released during the first phase of β-cell response normalized to the maximum increment, *ΔG*, of plasma glucose concentration after the injection (defined as the difference between the peak, *G*_*max*_, and the steady-state, *G*_*ss*_, value of plasma glucose concentration):

$$\Phi_{1}\;=\;\frac{CP_{0}}{\Delta G}$$ (A5)

The index *Φ*_*2*_ (min^-2^·(pmol/L C-peptide)/(mmol/L glucose)) describes the stimulatory effect of glucose concentration on provision into the β-cells and release of new insulin [18], thus:

$$\Phi_{2}\;=\;\frac{\partial^{2}\left(SR_{2}\left(t\right)\right)}{\partial G\partial t}\;=\;\frac{\partial^{2}\left(\gamma \cdot \left(G(t\right)-h\right)\cdot t)}{\partial G\partial t}\;=\;\gamma$$ (A6)

A steady-state responsiveness index, *Φ*_*ss*_ (min^-1^·(pmol/L C-peptide)/(mmol/L glucose)), can also be defined as the ratio between steady-state secretion rate, *SR*_*ss*_, and the end-test glucose concentration, *G*_*ss*_:

$$\Phi_{SS}\;=\;\frac{SR_{ss}}{G_{SS}}$$ (A7)

Total secretion rate, *TSR(t)* (min^-1^·pmol/L), is defined as the sum of the above steady-state and the steady-state secretion rates:

$$TSR\left(t\right)\;=\;SR\left(t\right)+SR_{ss}$$ (A8)

Integral of *TSR(t)* over the 90 min of our IVGTT yielded the total amount, *TIS*, of C-peptide secreted by the β-cells, per unit of distribution volume (pmol/L).

## Supporting Information

S1 Table Further Parameters of glucose metabolism in ZLR and ZFR groups. *S*_*G*_ (10^-2^ dL·kg^-1^·min^-1^), whole-body glucose effectiveness; *CP*_*0*_ (pmol/L) above steady-state C-peptide plasma concentration immediately after glucose injection; *SR*_*SS*_ (min^-1^·pmol/L), steady-state secretion rate; *TIS* (pmol/L) total amountof C-peptide secreted by the β-cells. All the parameters and indexes were estimated or computed by CPMM and IVGTT, except for *S*_*G*_, which was estimated by GKMM and IVGTT. The percent coefficient of variation of the estimates (CV%) was given in parentheses, when available.
(DOCX)
