# Supplementary material for: C-Peptide-Based Assessment of Insulin Secretion in the Zucker Fatty Rat: A Modelistic Study
Source: PLoS One. 2015 May 4;10(5):e0125252. doi: 10.1371/journal.pone.0125252 (PMC4418729; doi:10.1371/journal.pone.0125252)
Supplement: S1 Table — S G (10-2 dL·kg-1·min-1), whole-body glucose effectiveness; CP 0 (pmol/L) above steady-state C-peptide plasma concentration immediately after glucose injection; SR SS (min-1·pmol/L), steady-state secretion rate; TIS (pmol/L) total amountof C-peptide secreted by the β-cells. All the parameters and indexes were estimated or computed by CPMM and IVGTT, except for S G, which was estimated by GKMM and IVGTT. The percent coefficient of variation of the estimates (CV%) was given in parentheses, when available. (DOCX) [file pone.0125252.s001.docx]

**S1 Table.** Further Parameters of glucose metabolism in ZLR and ZFR groups.

| ZLR | *S_G_* | $CP_{0}$ | *SR_SS_* | *TIS* |
| --- | --- | --- | --- | --- |
| 1 | 9.16 (16.7) | 1494 (14.7) | 49.5 | 4.94 |
| 2 | 6.49 (12.9) | 2876 (14.3) | 25.7 | 4.51 |
| 3 | 5.12 (15.1) | 2003 (21.6) | 22.4 | 3.24 |
| 4 | 4.28 (35.8) | 2714 (14.8) | 33.2 | 4.46 |
| 5 | 6.72 (5.7) | 2961 (12.8) | 42.9 | 5.98 |
| 6 | 15.4 (10.2) | 2518 (29.0) | 93.6 | 9.99 |
| 7 | 7.0 (12.8) | 2351 (17.6) | 67.1 | 7.64 |
| ZFR | *S_G_* | $CP_{0}$ | *SR_SS_* | *TIS* |
| 1 | 5.15 (36.3) | 4383 (6.8) | 156 | 15.9 |
| 2 | 3.95 (35.8) | 5974 (8.8) | 276 | 29.2 |
| 3 | 3.50 (13.9) | 6391 (20.3) | 650 | 68.7 |
| 4 | 9.10 (9.5) | 5555 (19.9) | 780 | 76.1 |
| 5 | 5.97 (26.8) | 4272 (20.1) | 612 | 59.3 |
| 6 | 7.02 (22.1) | 4873 (19.5) | 609 | 59.7 |
| 7 | 3.74 (34.3) | 10813 (17.7) | 404 | 42.3 |

*S_G_* (10^-2^ dL·kg^-1^·min^-1^), whole-body glucose effectiveness; $CP_{0}$ (pmol/L) above steady-state C-peptide plasma concentration immediately after glucose injection; *SR_SS_* (min^-1^·pmol/L), steady-state secretion rate; *TIS* (pmol/L) total amountof C-peptide secreted by the β-cells. All the parameters and indexes were estimated or computed by CPMM and IVGTT, except for *S_G_,* which was estimated by GKMM and IVGTT. The percent coefficient of variation of the estimates (CV%) was given in parentheses, when available.
